# Kinematic Calibration of a Cable-Driven Parallel Robot for 3D Printing

**DOI:** 10.3390/s18092898

**Published:** 2018-09-01

**Authors:** Sen Qian, Kunlong Bao, Bin Zi, Ning Wang

**Affiliations:** School of Mechanical Engineering, Hefei University of Technology, Hefei 230009, China; qiansenhfut@126.com (S.Q.); baopinche@163.com (K.B.); wning@mail.hfut.edu.cn (N.W.)

**Keywords:** cable-driven parallel robot, kinematic calibration, error analysis, additive manufacturing

## Abstract

Three-dimensional (3D) printing technology has been greatly developed in the last decade and gradually applied in the construction, medical, and manufacturing industries. However, limited workspace and accuracy restrict the development of 3D printing technology. Due to the extension range and flexibility of cables, cable-driven parallel robots can be applied in challenging tasks that require motion with large reachable workspace and better flexibility. In this paper, a cable-driven parallel robot for 3D Printing is developed to obtain larger workspace rather than traditional 3D printing devices. A kinematic calibration method is proposed based on cable length residuals. On the basis of the kinematic model of the cable-driven parallel robot for 3D Printing, the mapping model is established among geometric structure errors, zero errors of the cable length, and end-effector position errors. In order to improve the efficiency of calibration measurement, an optimal scheme for measurement positions is proposed. The accuracy and efficiency of the kinematics calibration method are verified through numerical simulation. The calibration experiment based on the motion capture system indicates that the position error of end-effector is decreased to 0.6157 mm after calibration. In addition, the proposed calibration method is effective and verified for measurement positions outside optimal positions set through experiments.

## 1. Introduction

Cable-driven parallel robots (CDPRs) are known as a type of parallel robots. In CDPRs, the end-effector is suspended by several flexible cables, taking the place of rigid links in traditional rigid-link parallel robots [1,2,3]. Compared with traditional rigid-link parallel robots, CDPRs have much smaller inertia and higher payload to weight ratio, which provides high speed and acceleration of the end-effector [4]. In addition, due to the extension range and flexibility of cables, CDPRs can be applied in challenging tasks that require motion with large reachable workspace and better flexibility as well [5]. Three-dimensional (3D) printing technology has been greatly developed in the last decade and gradually applied in the construction, medical, and manufacturing industries. Limited workspace and accuracy restrict the development of 3D printing technology [6,7]. In this paper, a cable-driven parallel robot for 3D Printing is developed, in order to obtain larger workspace than traditional 3D printing devices.

The accuracy of CDPRs is mainly affected by the kinematic parameter errors. The kinematic parameter errors consist of the geometric parameter errors and the zero errors of the robot joints. It is necessary to compensate the geometric parameter errors and zero errors in order to realize the accurate position control of CDPRs [8]. The kinematic calibration is an effective method to improve the end-effector position accuracy by improving the precision of kinematic model. In general, the kinematic calibration can be divided into error modeling, error measurement, error identification, and error compensation [9,10,11]. The error measurement and error identification are two important steps in the calibration process. The error measurement is the process of measuring end-effector positions by means of the sensors, and comparing the measured values with the theoretical values. The error identification is a process of analyzing error measurement results based on a predetermined parameter identification equation, aiming to obtain kinematic parameter errors. Thus, the accuracy of the error identification is determined by error measurement, including the accuracy of measuring sensors and the selection of measuring positions. There are some areas in the workspace where the kinematic parameter errors have little influence on the end-effector position errors, while the influence of the measurement sensors noises on the accuracy of the error measurement is greatly increased. Therefore, the accuracy of error measurement can be improved obviously by selecting the end-effector positions with large position errors in the workspace as the measurement positions. According to the difference of measurement methods, the calibration methods can be divided into self-calibration method [12,13,14,15] and external calibration method [16,17,18,19].

In general, additional sensors in joints are necessary to obtain joint variables with self-calibration methods. In CDPRs, the end-effector is suspended by several flexible cables instead of rigid links in traditional rigid-link parallel robots, the installation of the sensors in robot joints will decrease the transmission accuracy of cables. Therefore, the external calibration method is more widely used for CDPRs. For the kinematic calibration of robots, many scholars have done a lot of research on improving the precision and efficiency of kinematic calibration, and proposed many efficient calibration methods. Joshi and Surianarayan [20] developed a six degrees of freedom cable-driven parallel device and proposed a kinematic calibration method based on the rotary angle errors of end platform. During the kinematic calibration, the assembly error of the inclinometers is considered and error angle on the perpendicularity of the two inclinometers is taken as the parameter to be calibrated. Ren et al. [21] has proposed a new orientation constraint that keeps the two orientation of the end-effector invariant, and deduces a calibration algorithm using different angle combinations. Lau et al. [22] has proposed a kinematic calibration method based on relative cable length measurement that is used to calibrate the initial cable length and end-effector poses of the CDPR. Duan et al. [23] employed the calibration of a cable-driven parallel manipulator by the six degrees of freedom laser tracker, meanwhile they verified the correctness and to what extent the calibration improves the system through the trajectory planning and motion control. Mustafa [24] developed a novel cable-driven manipulator with seven degrees of freedom, according to the characteristics of its redundant drives, a self-calibration scheme based on the cable joint variables is proposed.

The above calibration methods can achieve an effective calibration, however most of them need to measure a large number of redundant measurement poses in order to solve nonlinear problem in the calibration process. This is a very time-consuming operation process. Therefore, it is necessary to carry out the optimal selection of the measurement poses, which can reach the acceptable accuracy with fewer measurement configurations. The observability of the measurement configurations was used for this purpose. Borm and Menq [25] put forward the concept of observability index, which is used to evaluate the observability of the identification Jacobian matrix for kinematic errors, thus providing a theoretical basis for measurement positions optimization. Scholars have different opinions on the specific value of observability index. Borm and Menq [26] proposed the observability index based on the product of all non-zero singular values of identification Jacobian matrix. Driels and Pathre [27] proposed the condition number which equals the ratio of the largest singular value to the smallest non-zero singular value. Regarding the inverse of the condition number as the observability index, Nahvi et al. [28] regarded the minimum value of the singular value of the identification Jacobian matrix as the observability index. Jia et al. [29] proposed an optimization model with two observability indexes, which can take the partial features and the global ones of the singular values into account at the same time. With the observability index determined, the additional challenge is to find an effective algorithm to construct a configuration set whose observability index is maximal. Li et al. [30] and Zhou et al. [31] adopted DETMAX algorithm to construct the configuration set. Wang et al. [32] proposed the modified annealing algorithm for the polishing robot. These algorithms improved convergence performance but often led to a low convergence rate. Many recent methods used a large pool of randomly selected configuration candidates, and focused on finding a certain number of optimal configurations within the pool [33,34]. These methods usually make improvements on DETMAX algorithm. Because the size of the candidate pool is usually large, a huge number of computations is required to check all candidates in the pool. Therefore, many scholars put forward different methods to choose the smaller candidate pool. Li et al. [30] identified the candidate pool of a six DOF serial robot and compared the characteristics of the optimal selection schemes based on different observability indices. Nategh et al. [35] demonstrated the distribution of the candidate pool of a rigid parallel mechanism, and calibrated the six degrees of freedom rigid parallel mechanism using a minimum number of measurement configurations. Wang et al. [36] proposed an efficient configuration search method that is based on the closed-form mapping from configuration perturbations to singular-value variations. The above researches on the optimal selection of measurement poses provides a systematic implementation method for the optimal poses selection for parallel or serial rigid robots. Due to the unilateral actuating property of cables, some widely used calibration methods cannot be applied in CDPRs directly, which must be modified to meet the special property of cables.

In this paper, a cable-driven parallel robot for 3D Printing is developed. A kinematic calibration method is proposed based on cable length residuals. On the basis of the kinematic model of the cable-driven parallel robot for 3D Printing, the mapping model is established among geometric structure errors, zero errors of cable, and end-effector position errors. In order to improve the efficiency of calibration measurement, an optimal scheme for measurement positions is proposed. The accuracy and efficiency of the kinematics calibration method are verified through simulation. Furthermore, the calibration experiments based on the motion capture system are carried out to demonstrate the high performance and effectiveness of the proposed calibration method.

## 2. Description of Experimental Prototype

**Figure 1:**
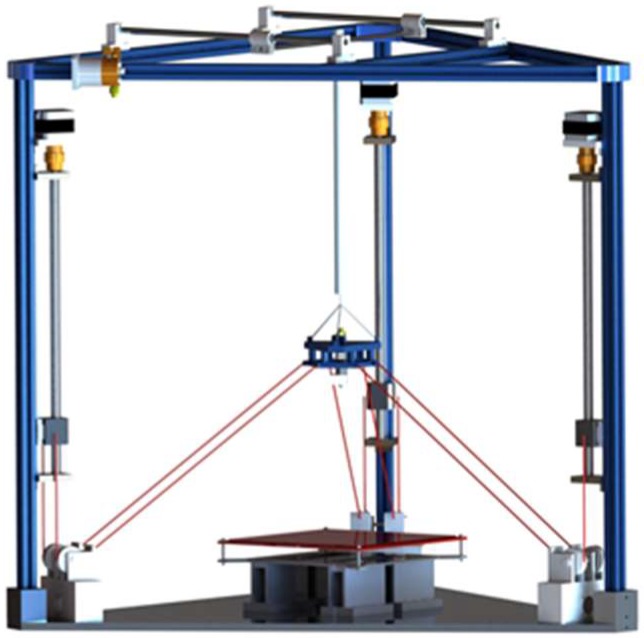
Three-dimensional (3D) model of the cable-driven parallel robot (CDPR) for 3D Printing.

In this paper, a novel CDPR for 3D printing is developed, which is designed for large-scale 3D printing. The structure of the CDPR for 3D Printing is shown in Figure 1. The extension range and flexibility of cables can significantly improve the workspace of 3D printing device at a lower manufacturing cost. The CDPR for 3D Printing adopts the Fused Deposition Molding (FDM) technology. The motion of the end-effector is mainly divided into scanning motion in each horizontal layer and lifting movement in vertical direction. Therefore, in order to realize 3D printing, the end-effector should have at least three degrees of freedom (3-DOF). At the same time, for the sake of ensuring the scanning accuracy of the device on each print layer, the rotational motion of the end-effector must be avoided and then the planeness of each printing layer can be ensured.

**Figure 2:**
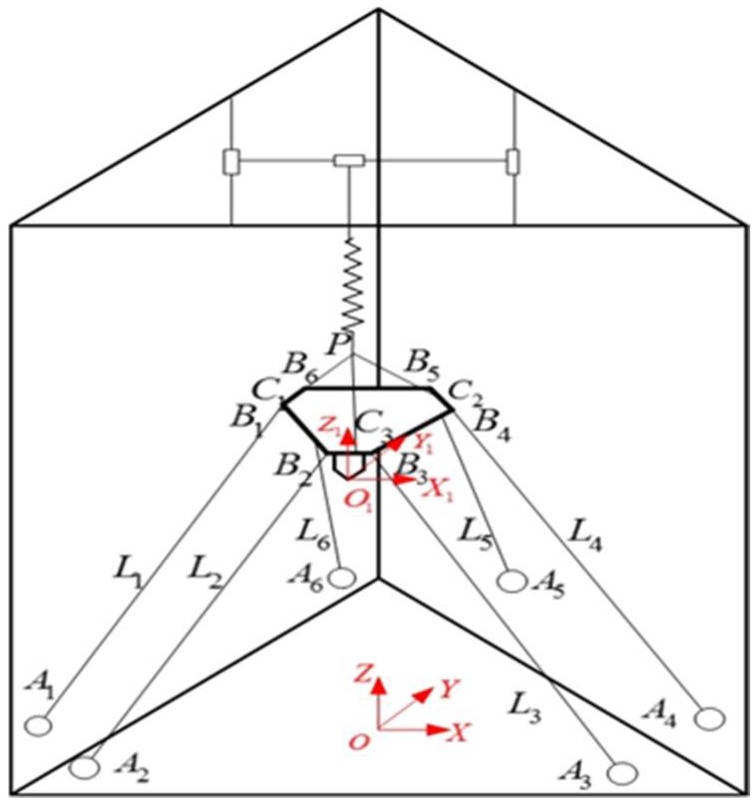
Sketch of the CDPR for 3D Printing.

Considering the above two requirements, the end-effector is driven by three cable groups. Each cable group consists of two parallel cables. A sketch of the CDPR for 3D Printing is shown in Figure 2. According to parallelogram principle, the end-effector cannot rotate around the coordinate axis under the constraint of three cable groups. Therefore, the end-effector only has three translational degrees of freedom, and the stable translation of the end-effector along each coordinate axis are realized under the constraint of the six cables and the follow-up spring, which is used to keep the tension on cables. The upper end of the follow-up spring is connected to the slide rail, which can guarantee that the follow-up spring is always in the vertical direction. Undoubtedly, the elasticity of the cables will affect the printing precision. It is necessary to choose the cables which has the high tensile strength. The modulus of the Kevlar49 cable is 861.9 cN/dtex and the modulus of the stainless steel cable is about 254.4 cN/dtex. Therefore, choosing Kevlar49 cables as the driven cables that will effectively reduce the effect of cable elasticity on printing accuracy.

As shown in Figure 2, one end ($B_{1}∼B_{6}$) of six cables ($L_{1}∼L_{6}$) is connected to the end-effector, and the cable winds around the fixed pulley set, through the cable outlets ($A_{1}∼A_{6}$), the other end is connected with the slider of the linear module which is made up of slide rail and ball screw. Three linear modules are used to pull six cables. The coordinate system of the CDPR for 3D printing is shown in Figure 2. OXYZ is the global coordinate frame which fixed to the center of the base, $OX_{1}Y_{1}Z_{1}$ is the local coordinate frame fixed to the end-effector.

## 3. Kinematic Error Modeling

### 3.1. Equivalent of Analytical Structure

**Figure 3:**
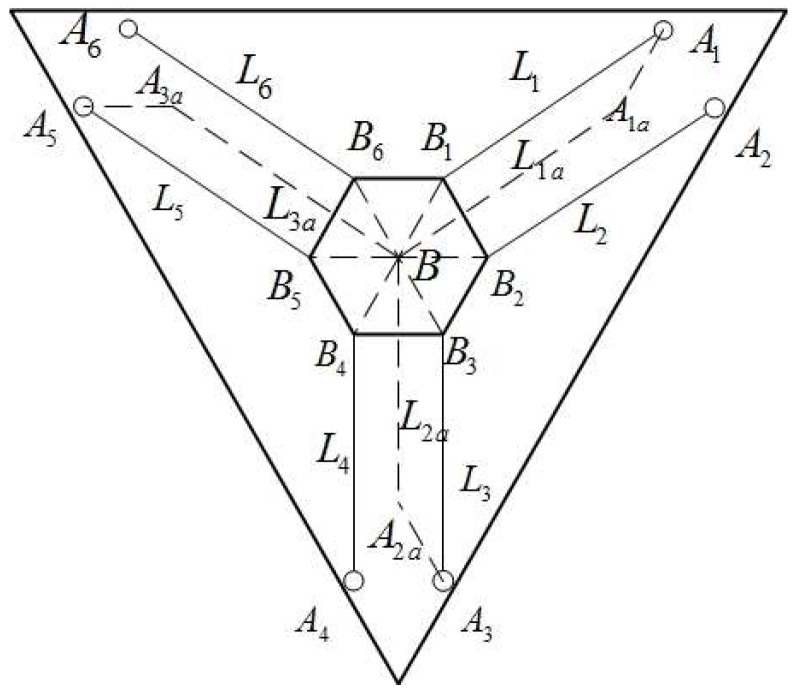
Schematic diagram of the equivalent model.

Before analyzing the kinematics, equivalent model of the CDPR for 3D printing can be established for simplicity. The schematic of the equivalent model is shown in Figure 3. For the presented 3-DOF CDPR, the center B of the end-effector is regarded as the equivalent end point. The six cables $L_{i}$_(*i* = 1, 2, 3, 4, 5, 6)_, can be equivalent to three cables $L_{ka}$ *_k_*_= (1, 2, 3)_, which facilitate the analysis of kinematic problems without loss of generality. First of all, vector $\vec{B_{i}B}$ are established through connecting the connection point $B_{i}$ between cables and end-effector to the centroid of the end-effector B. Due to the parallelogram principle, the orientation of the end-effector is always unchanged, therefore, the value of vectors $\vec{B_{i}B}$ are invariant, which is only related to the geometric structure of the end-effector. Secondly, in the context of guaranteeing vectors $\vec{A_{i}A_{ka}}$ are the same as vectors $\vec{B_{i}B}$, vectors $\vec{A_{i}A_{ka}}$ are established by connecting the cable outlets *A*_1_, of fixed pulley to virtual cable outlets $A_{ka}$. Therefore, it can be obtained from the principle of parallelogram that: $L_{1a}$//$L_{1}$//$L_{2}$, $L_{i}$//$L_{3}$//$L_{4}$, $L_{3a}$//$L_{5}$//$L_{6}$. According to the above parallel conditions, we can simplify the kinematic inverse operation of six cables to the kinematic inverse operation of three cables without losing the generality, and then the computational complexity of inverse kinematic analysis is greatly reduced.

### 3.2. Kinematic Error Modeling

There are several factors that reduce the printing accuracy of the CDPR for 3D Printing, including kinematic error, transmission error, nonlinear error of the control system, deformation error, the measurement error of the sensors, and thermal error. The kinematic errors caused by the manufacturing and assembly are the main error sources. The error model is established in order to describe the relationship between the position errors of the end-effector, the geometric parameter errors of the mechanism, and the zero errors of the cable length.

**Figure 4:**
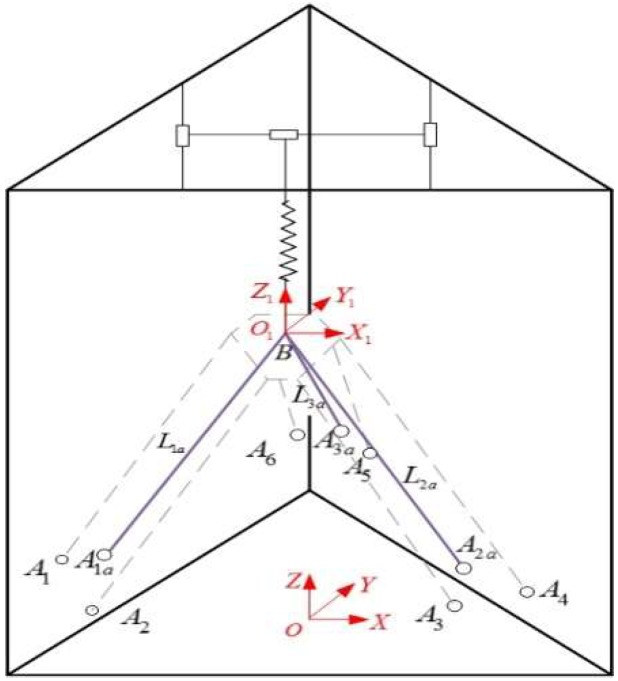
Equivalent schematic diagram of the CDPR for 3D Printing.

Kinematic analysis is carried out based on the equivalent model shown in Figure 4. B(x,y,z) can be represented as the reference point of the local coordinate frame. The Euler angle of the moving coordinate frame is $\left[\begin{array}{ccc} 0 & 0 & 0 \end{array}\right]$, the global coordinates of the virtual cable outlets $A_{ka}$ are represented as [$x_{ka},y_{ka},z_{ka}$], therefore, the length of cables can be obtained as follows:

(1) $$L_{ka}=L_{ko}+L_{kr}=\sqrt{(x-x_{ka}{)}^{2}+{\left(y-y_{ka}\right)}^{2}+{\left(z-z_{ka}\right)}^{2}}$$

where $L_{ka}$ are the cable lengths from virtual cable outlets to equivalent end point, B, $L_{ko}$ are the cable lengths from virtual cable outlets to the equivalent end point B at the initial position, $L_{kr}$ are cable lengths variable, which are defined as the differences between $L_{ko}$ and $L_{ka}$.

The implicit function form of Equation (1) can be written as:

(2) $$f_{k}(x,y,z,x_{ka},y_{ka},z_{ka},L_{ko}+L_{kr})=0$$

One can obtain from Equation (2) that the kinematic errors of the device consist of the position error of virtual cable outlets that is represented as $\left(\begin{array}{ccc} dx_{ka} & dy_{ka} & dz_{ka} \end{array}\right)$, and the error of initial cable length (i.e., the zero errors of the cable length) that can be represented as $dL_{k}$, *_k_*_= (1, 2, 3)_. Thus, a total of 12 geometric error sources can be obtained. 

Equation (2) can be differentiated to obtain the following equation:

(3) $$\begin{array}{c} 2(L_{ko}+L_{kr})dL_{k}-2(x-x_{ka})dx+2(x-x_{ka})dx_{ka}-2(y-y_{ka})dy\\ +2(y-y_{ka})dy_{ka}-2(z-z_{ka})dz+2(z-z_{ka})dz_{ka}=0 \end{array}$$

Equation (3) can be expanded and written into matrix form. The mapping relationship of geometric parameter errors, zero errors of the cable length and the end-effector position errors can be obtained as follows:

(4) $$C\delta =D^{T}\Delta q$$

where:

$$\delta ={\left(\begin{array}{ccc} dx & dy & dz \end{array}\right)}^{T}$$

$$\begin{array}{c} \Delta q=\\ {\left(\begin{array}{ccc} \begin{array}{ccc} dx_{1a} & dy_{1a} & dz_{1a} \end{array} & \begin{array}{ccc} dx_{2a} & dy_{2a} & dz_{2a} \end{array} & \begin{array}{ccc} dx_{3a} & dy_{3a} & \begin{array}{ccc} dz_{3a} & dL_{1} & \begin{array}{cc} dL_{2} & dL_{3} \end{array} \end{array} \end{array} \end{array}\right)}^{T} \end{array}$$

$$C=\left(\begin{array}{ccc} x-x_{1a} & y-y_{1a} & z-z_{1a}\\ x-x_{2a} & y-y_{2a} & z-z_{2a}\\ x-x_{3a} & y-y_{3a} & z-z_{3a} \end{array}\right)$$

$$D=\left(\begin{array}{ccc} x-x_{1a} & 0 & 0\\ y-y_{1a} & 0 & 0\\ z-z_{1a} & 0 & 0\\ 0 & x-x_{2a} & 0\\ 0 & y-y_{2a} & 0\\ 0 & z-z_{2a} & 0\\ 0 & 0 & x-x_{3a}\\ 0 & 0 & y-y_{3a}\\ 0 & 0 & z-z_{3a}\\ L_{1o}+L_{1r} & 0 & 0\\ 0 & L_{2o}+L_{2r} & 0\\ 0 & 0 & L_{3o}+L_{3r} \end{array}\right)$$

where δ is the position error of the end-effector, and Δq is kinematic error of the CDPR for 3D printing.

The matrix C are reversible in nonsingular positions, thus, Equation (4) can be rewritten as follows:

(5) $$\delta =J_{0}\Delta q$$

where

$$J_{0}=C^{-1}D^{T}$$

where $J_{0}$ is the identification Jacobian matrix.

## 4. Optimal Selection

During the process of measuring position, the noise of the measurement sensors is inevitable. The observability is considered as a criterion to find out optimal measurement positions where the position errors of the end-effector are most distinguishable. The noise of the measurement sensors slightly affect the error measurement results in optimal measurement positions.

Optimal selection for measurement positions in this section proceeds in two stages. Through analyzing the position errors distribution in the workspace of the CDPR for 3D printing, the preliminary area of the measurement positions are determined. The coordinates of each optimal position are accurately determined through the calculating the observable index of each point in the preliminary selection region.

### 4.1. Analysis of Workspace and Preliminary Selection 

The workspace of the CDPR for 3D printing is the position set that the end-effector can reach within the structural frame. An important characteristic of CDPRs is well known, as cables can only be driven by positive tension in order to keep the straight line shape, rather than negative compression. Therefore, the constraint should be satisfied that cables are all in tension ($t_{k}>0$) in the process of calculating the workspace. 

Due to the low inertia of the CDPRs rather than rigid link robots, the end-effector can obtain higher acceleration. Therefore, the dynamic constraints caused by acceleration should be taken into account in the analysis of the workspace. The specific mathematical description of acceleration constraints is as follows:

(6) $$\begin{array}{cc} \forall a<a_{\max} & \exists T>0:A\cdot T+W \end{array}=ma\cdot c$$

where $a_{\max}$ is the scalar value of the maximum acceleration of the end-effector, $A=\left(\begin{array}{ccc} u_{1} & u_{2} & u_{3} \end{array}\right)$ is the structure matrix of the robot, $u_{k}$ *_k_*_= (1, 2, 3)_ is unit directional vector of each cable, and $T={\left(t_{1},t_{2},t_{3}\right)}^{T}$ is the tension value of each cable, W is the external force on the end-effector, the value of which is about 15 N, m is the weight of end-effector, a is the acceleration scalar for end-effector, and c is the unit directional vector of end-effector acceleration.

As described in Section 3, the motion of the end-effector is equivalent to the movement under the constraint of three cables and the follow-up spring. The preload of the follow-up spring is far greater than the self-weight of the end-effector. The follow-up spring can be regarded as a special cable whose tension changes with the z value of the end-effector. The direction of the spring is along the z axis of the global coordinate. Therefore, the force balance equation of the CDPR for 3D printing can be expressed as follows:

(7) $$A\cdot T+k(z_{k}-z)\cdot b-mg\cdot b=ma\cdot c$$

where $b={\left(\begin{array}{ccc} 0 & 0 & 1 \end{array}\right)}^{T}$ is the unit directional vector of the follow-up spring tension, k is the elastic coefficient of the follow-up spring, $z_{k}$ is the z value of the end-effector in the initial state, Z is the z value of the end-effector, and g is the acceleration of gravity.

Equation (7) can be rewritten as follows:

(8) $$A^{\prime }T^{\prime }=ma\cdot c$$

where

$$A^{\prime }=(\begin{array}{cc} A & b \end{array})$$

$$T^{\prime }=\left(\begin{array}{c} T\\ k(z_{k}-z)-mg \end{array}\right)$$

According to Equation (8), the mathematical description of acceleration constraints can be rewritten as follows:

(9) $$\begin{array}{cc} \forall a<a_{\max} & \exists T^{\prime }>0:A^{\prime }\cdot T^{\prime } \end{array}=ma\cdot c$$

The solution of equation can be expressed as:

(10) $$T^{\prime }=A^{\prime +}\cdot ma\cdot c+X+{\left(\begin{array}{ccc} ma & ma & ma \end{array}\right)}^{T}$$

where $A^{\prime +}$ is generalized inverse matrix of $A^{\prime }$, $X+{\left(\begin{array}{ccc} ma_{\max} & ma_{\max} & ma_{\max} \end{array}\right)}^{T}$ is a vector in null space of $A^{\prime }$, according to matrix theory, $r\cdot [X+{\left(\begin{array}{ccc} ma_{\max} & ma_{\max} & ma_{\max} \end{array}\right)}^{T}]$ is still a vector in null space of $A^{\prime }$, while r is an arbitrary constant, therefore, Equation (10) can be transformed into:

(11) $$T^{\prime }=ma\cdot [A^{\prime +}\cdot c+r\cdot {\left(\begin{array}{ccc} 1 & 1 & 1 \end{array}\right)}^{T}]+r\cdot X$$

The inertial force generated by the acceleration in specific direction will produce a large resolution force on the cables. Hence, it is necessary to apply the solution method of the force-closure workspace [37] to the solution of the workspace that is constrained by the acceleration. It can be obtained from Equation (11) that if ∃X>0, $T^{\prime }>0$ can be satisfied while r trend to be infinite. According to matrix theory, if $\exists V\in \ker(A^{\prime })$, ∃X>0 can be satisfied while V>0. Therefore, the solution conditions of the workspace can be expressed as:

(12) $$\exists V\in \ker(A^{\prime }):V>0$$

According to the need of actual printing, in addition to guaranteeing the tension of cables are all under acceleration constraints, the following constraints should be satisfied during the simulation:

(1) The rigid frame constraint on the range of the workspace. Specific constraints can be expressed as follows:

(13) $$\{\begin{array}{c} -l_{0}/2<x<l_{0}/2\\ -l_{0}\tan(\pi /6)<y<\sqrt{3}(l_{0}/2-\left|x-l_{0}/2\right|)\\ 0<z<l_{z} \end{array}$$

where $l_{0}$ is the side length of a regular triangle with three virtual cable outlets as apexes, and $l_{z}$ is the initial height of the end-effector. 

(2) The cable length constraints on the range of the workspace. The device is driven by the fixed length cables. When the end-effector is in the initial position, the cable length from the cable outlets to the end-effector is the maximum $l_{\max}$. The length of each cable at the remaining positions is less than or equal to $l_{\max}$. Hence, the specific constraints can be expressed as follows:

(14) $$0<L_{ka}<l_{\max}$$

(3) The constraint of cable inclination relative to the horizontal plane. When the inclination of the cables $\alpha_{k}$ *_k_*_= (1, 2, 3)_ is too small, it can be known from the decomposition of the force that the force on each cable will increase dramatically. Too large cable tension will affect the performance of the motors. The specific constraints are expressed as follows:

(15) $$\{\begin{array}{c} 0<\alpha_{1}<\pi /12\\ 0<\alpha_{2}<\pi /12\\ 0<\alpha_{3}<\pi /12 \end{array}$$

**Figure 5:**
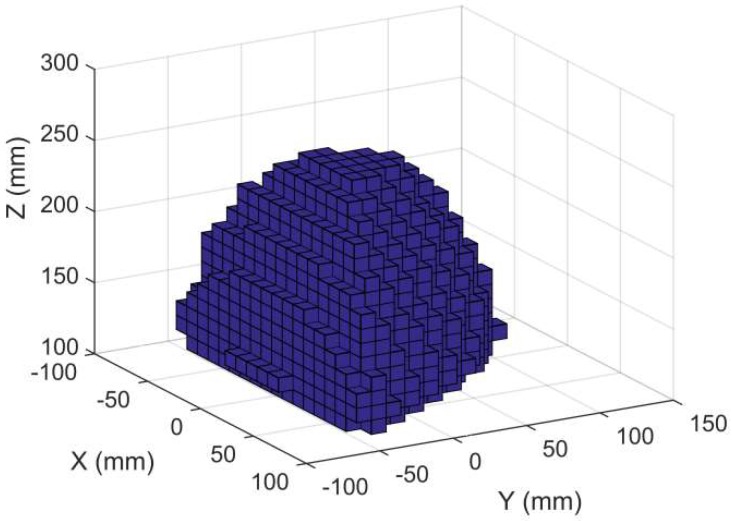
The workspace of the CDPR for 3D Printing.

As shown in Figure 4, the whole frame can be sketched as a tri-prism structure. The bottom of the tri-prism is a regular triangle with the side length 600 mm, and the height of the tri-prism is 650 mm. According to the constraints and structural parameters listed above, the workspace of the end-effector is obtained as Figure 5:

The shape of the workspace can be sketched as a tri-prism. The bottom of the tri-prism is a regular triangle with the side length 180 mm and the height of the tri-prism is 240 mm. During the experimental stage, in order to ensure the stability of the CDPR for 3D Printing, the CDPR for 3D Printing is driven by the fixed length cables. However, it results in a smaller workspace. As mentioned above, the cable length and the rigid frame are the main constraints of the workspace. Therefore, the workspace can be expanded through adopting the winches to drive the CDPR for 3D printing or enlarging the scale of the rigid frame. Due to the extension range and flexibility of cables, the workspace can be expended at a low engineering cost.

**Figure 6:**
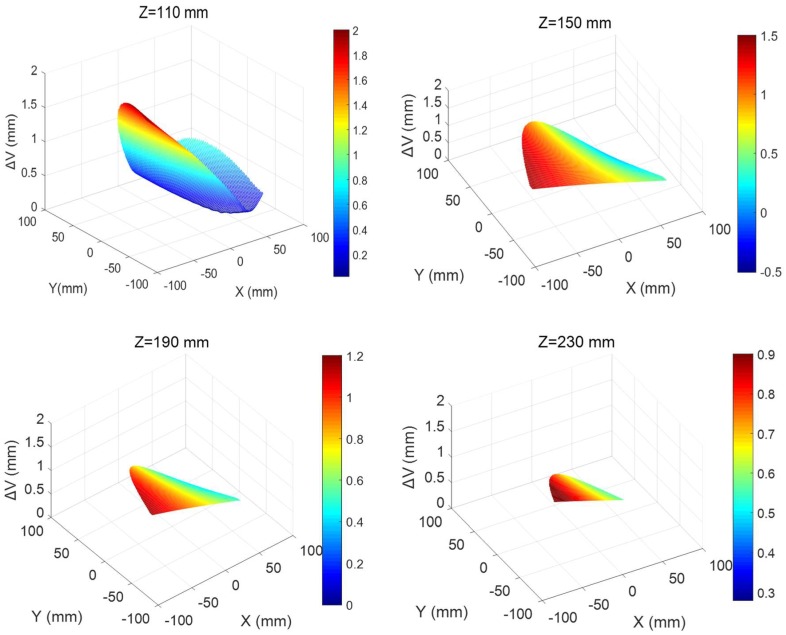
The workspace of the CDPR for 3D Printing.

In order to reduce the search range of optimal measurement positions and obtain the preliminary positions with greater observability, it is necessary to analyze the distribution of position errors in the workspace. Firstly, random geometric errors (from −1 mm to 1 mm) is added to all desired kinematic parameters. The construction volume errors of the end-effector in the workspace are obtained via forward kinematics. The construction volume error of parallel 3D printing device at different height in workspace can be obtained, the results are depicted in Figure 6. The construction volume errors in the workspace of the CDPR for 3D printing can be expressed as follows:

(16) $$\Delta V=\sqrt{\Delta x^{2}+\Delta y^{2}+\Delta z^{2}}$$

where Δx is the position error of end-effector in x direction, Δy is the position error of end-effector in y direction, and Δz is the position error of end-effector in z direction.

As shown in Figure 6, the z values of the end-effector are 110 mm, 150 mm, 190 mm, and 230 mm, respectively. It can be seen that the maximum value of the construction volume errors is distributed at the boundary of the each horizontal plane and the structural volume error of the end-effector decreases with the printing height. Based on the principle of selecting the positions with the larger structural volume errors, the boundary region of the z = 110 mm plane in the workspace is regarded as the preliminary selection area of the measurement positions.

### 4.2. Optimal Positions Selection

After the preliminary selection area of the measurement positions is determined, the optimal positions selection can be divided into two steps. Firstly, the candidates of measurement positions should be selected from z = 110 mm. Secondly, an effective algorithm should be applied to construct an optimal position set, the observability index of which is maximal. 

**Figure 7:**
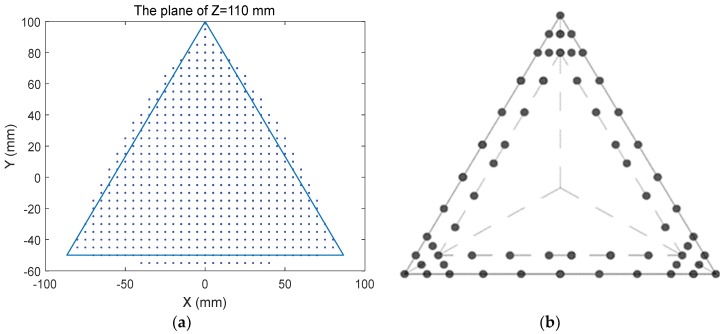
The distribution of candidate points: (**a**) The planform of Z = 110 mm; (**b**) Discretized boundary of the Z = 110 mm.

As shown in Figure 7a, the z = 110 mm plane in the workspace is an equilateral triangle region with sides equal to 170 mm. In order to facilitate the selection of the candidates of measurement positions, the boundary of the equilateral triangle region is discretized. Through moving the sides of the equilateral triangle inward by 1mm, an embedded equilateral triangle can be obtained. The candidates of measurement positions are distributed along the edges of the two equilateral triangles. The distribution of the position candidates is shown in Figure 7b, 60 candidates of measurement positions are selected.

As mentioned in Section 3.2, the CDPR for 3D printing has 12 independent kinematic parameter errors, and each measurement position has 3 kinematic constraint equations. In order to accomplish the error identification, the number of measurement positions K is at least 4. Therefore, the optimal selection of the measurement positions in this paper aimed at selecting 4 optimal positions with the maximum observability index from the 60 candidate positions. Four kinds of observability index are mentioned in the introduction. In this paper, the reciprocal of conditional numbers $O_{2}$, is chosen as the observability index. Among four kinds of observability index, the calibration algorithms will be led to a high convergence rate while regarding $O_{2}$ as observability index [30]. Regarding the non-zero singular value of the identification Jacobian $J_{0}$, as $\sigma_{m}\leq \ldots \leq \sigma_{1}$, the observability index $O_{2}$, can be expressed as:

(17) $$O_{2}=\sigma_{m}/\sigma_{1}$$

where $\sigma_{m}$ is the maximal non-zero singular value of the identification Jacobian, and $\sigma_{1}$ is the minimal non-zero singular value of the identification Jacobian.

By calculating the observability index $O_{2}$, of the identification Jacobian $J_{0}$, in the above 60 candidate positions, the K(K = 4) positions with the maximum observability index $O_{2}$, among the 60 candidate positions are selected. The method of optimal positions selection is presented as follows:

(1) $Q_{c}$ is the candidate position set, there are C = 60 candidate points in the initial state.

(2) $N_{l}$ is the optimal measurement positions set, it concludes a number of l = 0 positions in the initial state.

(3) Search the $O_{2}$ maximum point $q_{l}$, in $Q_{c}$, using the extremum seeking function.

(4) Add $q_{l}$ to $N_{l}$, l=l+1, meanwhile, remove $q_{l}$ from $Q_{c}$, C=C−1

(5) Repeat steps 3, 4, until l=K, stop the cycle.

After the above steps, the result is that the maximum observation index of the optimal positions set is $O_{2\max}$ = $13.99\times 10^{2}$, the coordinates of each optimal position in $N_{l}$ are shown in Table 1.

## 5. Simulation

As mentioned above, the coordinates of the cable outlets and the initial cable length are the main kinematic parameters that determine the printing accuracy of the CDPR for 3D printing. In order to improve the printing accuracy, a kinematic calibration method based on cable length residuals is proposed. Through measuring the end-effector position errors and cable length variables synchronously by the motion capture system, the calibration method can calibrate the coordinates of the cable outlets and the initial cable length. This section mainly focuses on the simulation for the calibration to verify the effectiveness of this method.

### 5.1. Error Identification Model

The main aim of kinematic calibration is to reduce the position errors of end-effector by the way of kinematic parameters compensation. Therefore, it is the key problem of kinematic calibration to establish the error identification model. In this paper, the functional formula for the error identification is established, which realizes the error identification among the position errors of end-effector, geometric parameter errors, and zero errors of cable length.

The difference between the measured and actual values of the end-effector positions can be expressed as follows:

(18) $$e_{k}=e_{m}-e$$

where $e_{k}$ is the residuals between the measured and the true position coordinates of the end-effector, that is, the measurement noise of the measurement sensor, $e_{m}$ is the measured position coordinates of the end-effector, and e is the true position coordinates of the end-effector.

According to Equation (1), the kinematic constraint equation of the CDPR for 3D printing is as follows:

(19) $$L_{ka}=L_{ko}+L_{kr}=\sqrt{(x-x_{ka}{)}^{2}+{\left(y-y_{ka}\right)}^{2}+{\left(z-z_{ka}\right)}^{2}}$$

where

$$L_{ko}={L_{ko}}^{\prime }+\Delta l_{k},\;L_{kr}={L_{kr}}^{\prime }+\psi_{r},\;x=x^{\prime }+\Delta x+e_{kx}$$

$$y=y^{\prime }+\Delta y+e_{ky},\;z=z^{\prime }+\Delta z+e_{kz},\;x_{ka}={x_{ka}}^{\prime }+\Delta x_{ka}$$

$$y_{ka}={y^{\prime }}_{ka}+\Delta y_{ka},\;z_{ka}={z^{\prime }}_{ka}+\Delta z_{ka},\;e=e^{\prime }+\delta ,\;q=q^{\prime }+\Delta q$$

where ${L_{ko}}^{\prime }$ and $\Delta l_{k}$ are nominal values and zero errors of initial cable length, respectively. ${L_{kr}}^{\prime }$ and $\psi_{kr}$ are measurement values and measurement errors of the cables length, $x^{\prime }$ and Δx are the nominal values and errors of x coordinates of the end-effector, respectively. $e_{kx}$ is the measurement noise in x direction of measurement sensors. $y^{\prime }$ and Δy are the nominal values and errors of y coordinates of the end-effector, respectively. $e_{ky}$ is the measurement noise in y direction of measurement sensors. $z^{\prime }$ and Δz are the nominal values and errors of z coordinates of the end-effector, respectively. $e_{kx}$ is the measurement noise in z direction of measurement sensors. ${x_{ka}}^{\prime }$ and $\Delta x_{ka}$ are the nominal values and errors of x coordinate of each cable outlet, respectively. ${y^{\prime }}_{ka}$ and $\Delta y_{ka}$ are the nominal values and errors of y coordinate of each cable outlet, respectively. ${z^{\prime }}_{ka}$ and $\Delta z_{ka}$ are the nominal values and errors of z coordinate of each cable outlet, respectively. $e^{\prime }$ and δ are the end-effector nominal position parameters set and the position parameter errors set, respectively. $q^{\prime }$ and Δq are nominal kinematic parameters set and kinematic errors set, respectively.

The error identification model is expressed as:

(20) $$\delta =f\left(L_{kr},q^{\prime }+\Delta q\right)-(e^{\prime }+e_{k})$$

where f(x) is the forward kinematics equation of the CDPR for 3D printing. 

Equation (20) can be rewritten as a functional form.

(21) $$e_{k}=f\left(L_{kr},\;q^{\prime }+\Delta q\right)-e^{\prime }-\delta$$

For the CDPR, the function equation based on the cable length residual is more convenient for the implementation of the nonlinear least square method. Hence, the functional equation for error identification is rewritten as follows:

(22) $$\begin{array}{c} \zeta_{i}={L_{ko}}^{\prime }+\Delta l_{k}+{L_{kr}}^{\prime }\\ -\sqrt{(x^{\prime }+\Delta x-x_{ka}^{\prime }-\Delta x_{ka}{)}^{2}+{\left(y^{\prime }+\Delta y-y_{ka}^{\prime }-\Delta y_{ka}\right)}^{2}+{\left(z^{\prime }+\Delta z-z_{ka}^{\prime }-\Delta z_{ka}\right)}^{2}} \end{array}$$

where $\zeta_{i}$ is a set of cable length residuals caused by the measurement noise of the measurement sensors. 

The error identification problems are usually solved through the nonlinear least square method. Hence, the equation of error identification can be written as follows:

(23) $$F=\underset{\left\{\Delta l_{k},(\Delta x_{ka},\Delta y_{ka},\Delta z_{ka})\right\}}{\min}\sum_{i=1}^{n}{\zeta_{i}}^{T}\zeta_{i},k\in \{1,2,3\}$$

### 5.2. Simulation Verification 

The specific simulation calibration process is as follows:

(1) The optimal measurement positions set $N_{l}$, is regarded as the measurement positions set. The solving of inverse kinematic equation is performed based on the nominal values of the measurement positions. The cable length $L_{kr,l}$, corresponding to the each measurement position can be obtained, which is used to simulate the cable length measured by motion capture system.

(2) By substituting the cable length $L_{kr,l}$, into the forward kinematic model based on the true values of geometric parameters, the actual coordinates $e_{l}$, of each measurement position will be calculated. The true values of the geometric parameters contain the true coordinate values of the cable outlets and the true values of the initial cable length, which are given ideal parameter during the simulation. The main purpose of this step is to simulate the measurement value of the motion capture system in the actual calibration process.

(3) The position errors of end-effector $\delta_{i}$, are calculated and substituted into the error identification equation Equation (23). The error identification values $\Delta q_{i}$, are calculated and compensated to each kinematic parameter $q_{i}^{\prime }=q_{i}^{\prime }+\Delta q_{i}$, i=i+1 and the new nominal value, $q_{i}^{\prime }$, of each parameter is obtained.

(4) Compare the absolute value of $\Delta q_{i}$ with the given threshold ε, if $\Delta q_{i}\leq \varepsilon$, the simulation calibration is terminated, otherwise i=i+1, return to step (1).

Because the motion control system is based on the equivalent model of the CDPR for 3D printing mentioned in Section 3, the process of simulation calibration is based on the equivalent kinematics model. The true coordinates of the three virtual cable outlets are $a_{1}$ = (−260, −150.111, 78), $a_{2}$ = (260, −150.111, 78), and $a_{3}$ = (0, 300.222, 78), respectively, which are given ideal parameter during the simulation. The initial position of end-effector is specified as (0, 0, 330), the true values of the initial cable length can be determined using inverse kinematics as $L_{ko}$ = 392 mm, (*k* = 1, 2, 3). Before the kinematic calibration of the CDPR for 3D printing, preliminary measurement should be performed to obtain the nominal values of the kinematic parameters. According to the existing measuring tools, the errors of the preliminary measurement are generally within 3 mm. In the simulation, random errors in the range of ±3 mm are added to the true values of geometric parameters to obtain the nominal values of kinematic parameters. Therefore, it is assumed that the nominal values of the initial cable length are $L_{1o}^{\prime }$ = 390 mm, $L_{2o}^{\prime }$ = 391.5 mm, and $L_{3o}^{\prime }$ = 389 mm, respectively. The nominal coordinates of each cable outlet are $a_{1}^{\prime }$ = (−258, −149, 79), $a_{2}^{\prime }$ = (263, −148, 77), and $a_{3}^{\prime }$ = (0, 301, 78.5), respectively.

Based on the data listed above, the threshold is set as ε = ${1\times 10}^{-4}$ mm, the simulation calibration of a CDPR for 3D printing is performed. After the parameters iteration, the termination condition is satisfied while the parameter identification time is i = 4. The specific simulation results are shown in Table 2.

**Figure 8:**
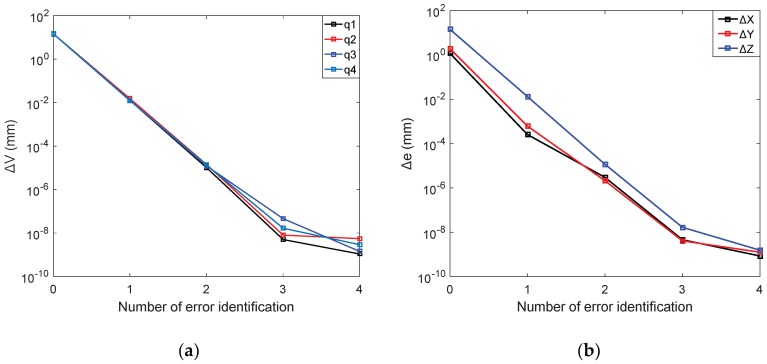
The relationships between errors and identification times: (**a**) The change of construction volume errors; (**b**) The change of position errors.

As shown in Figure 8a, before the calibration, the maximum construction volume error in the optimal measurement positions set is $\Delta V_{0\max}$ = 14.4223 mm. After four times error identification, the maximum structural volume error in the optimal measurement positions set is $\Delta V_{4\max}$ = $5{.4321\times 10}^{-9}$ mm.

The position errors in x direction are set as $\Delta x_{l}$, the average absolute value of the position errors in *x* direction can be expressed as $\Delta e_{x}=\sum_{l}^{4}|\Delta x_{l}|/4$,which is used as a standard to study the position errors in *x* direction of the end-effector. Similarly, the position errors of *y* and *z* axis are measured as $\Delta e_{y}$ and $\Delta e_{z}$. Figure 8b shows the variation of the $\Delta e_{x}$, $\Delta e_{y}$, and $\Delta e_{z}$ with the error identification times. It can be seen from Figure 8b that before calibration, the average absolute values of the position errors in *x*, *y*, and *z* directions are $\Delta e_{x0}$ = 1.1772 mm, $\Delta e_{y0}$ = 1.9062 mm, and $\Delta e_{z0}$ = 14.1970 mm, respectively. After four times error identification, the mean absolute value of the position errors in the direction of each coordinate axis are reduced to $\Delta e_{x4}$ = ${8.4842\times 10}^{-10}$ mm, $\Delta e_{y4}$ = ${1.2539\times 10}^{-9}$ mm, and $\Delta e_{z4}$ = ${1.5540\times 10}^{-9}$ mm. The experimental results show that this calibration method is of fast convergence speed and favorable calibration accuracy.

## 6. Calibration Experiment

**Figure 9:**
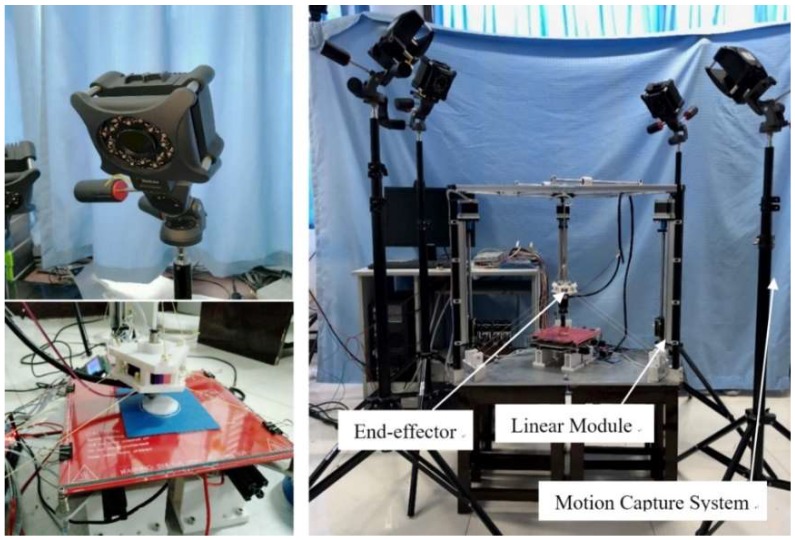
Experimental device of kinematic calibration.

The experimental platform of the CDPR for 3D printing is shown in Figure 9, the stepping motor drives the linear module to drive the fixed-length cables to realize the movement of the end-effector. Therefore, the displacements of the sliders on the linear module is equal to the change of cable length. The coordinates of the markers attached to the sliders can be measured with the motion capture system.

### 6.1. Measurement

Before calibrating the coordinate values of each cable outlet and the initial cable length accurately, the initial measurement is carried out in order to obtain the reasonable structural parameters required by the control system. The upper surface of the base is the plane (*z* = 0 mm), the centroid of the regular triangular base is regarded as the origin of the global coordinate frame. The coordinate values of the cable outlets and the initial cable length measured in the global coordinate frame are shown in Table 3.

As mentioned in Section 5.2, the coordinates of the cable outlets that should be calibrated are the coordinates of the three virtual cable outlets. According to the measured coordinates of each cable outlet, the coordinate values of virtual cable outlets are calculated based on the equivalent principle described in Section 3.1. The length of each cable group is the same as that of the corresponding virtual cables. The specific coordinate values of virtual cable outlets are shown in Table 4.

**Figure 10:**
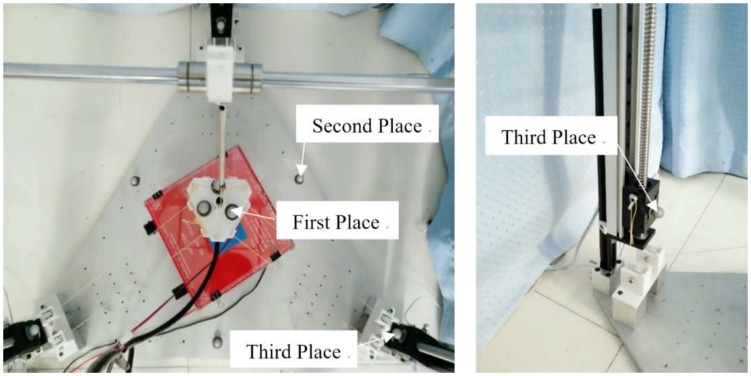
The layout of the markers.

Before the measurement, the markers should be adhere to the prototype to obtain the positions of each mobile component. The markers are distributed in three places, as shown in Figure 10. The first maker place is on the end-effector where the markers are triangle-distributed. The second maker place is triangle-distributed on the base. The centroid of the equilateral triangle is the coordinate origin of the global coordinate frame. The third maker place is located on the sliders of linear modules, the differences between the *z* values of the markers measured by the motion capture system and the *z* values of markers in the initial state are the cable length variables.

During the data measurement, the end-effector is moved to the above optimal positions (*z* = 110 mm), respectively. Meanwhile, the actual coordinate value of each position and the cable length are measured by the motion capture system synchronously.

### 6.2. Data Processing

In this calibration experiment, the measurement rate of the motion capture system is 60 frames per second. The effective test time for each location is 3 s. The coordinate of the markers is based on the world coordinate system determined by self-calibration of the motion capture system. However, the world coordinate system and the global coordinate system of the experimental device are difficult to be coincident though artificial setting. Therefore, in order to apply the measurement data to the error identification, the coordinate values measured by the motion capture system should be converted first.

The coordinate system A is set as the world coordinate system, the coordinate system B is set as the global system of experimental device, and the coordinate origin of coordinate system B in the coordinate system A is ${}^{A}p_{B0}$. Through determining ${}^{A}p_{B0}$ and *y* axis direction vector of the coordinate system B in the coordinate system A, the conversion relationship between the two coordinate systems can be determined.${}^{A}p_{B0}$ is the centroid coordinate values of the regular triangle determined by the markers on the base, the *y* axis direction vector of the coordinate system B is also determined by the three attached markers on the base, which is the unit direction vector from a marker to the origin of coordinate system B. After obtaining the measurement data as described above, the rotation matrix ${}_{B}^{A}R$ between the coordinate system A and the coordinate system B can be calculated out. The coordinate values of the point p that is measured by the motion capture system in coordinate system A is ${}^{A}p$, the position of point p in coordinate system B is as follows:

(24) $${}^{B}p={}_{B}^{A}R^{-1}({}^{A}p-{}^{A}p_{B0})$$

where ${}_{B}^{A}R^{-1}$ is the inverse matrix of rotation matrix ${}_{B}^{A}R$.

After the data measurement, the coordinate values of three markers (F1, F2, F3) on the base in the coordinate system B are shown in Table 5. According to the data, it is calculated that ${}^{A}p_{B0}$ is (542.4536, 48.4085, 23.1651) and the specific value of rotation matrix ${}_{B}^{A}R$ is as follows:

(25) $${}_{B}^{A}R=\left[\begin{array}{ccc} 0.483768011 & 0.875196271 & 0\\ -0.875196271 & 0.483768011 & 0\\ 0 & 0 & 1 \end{array}\right]$$

**Figure 11:**
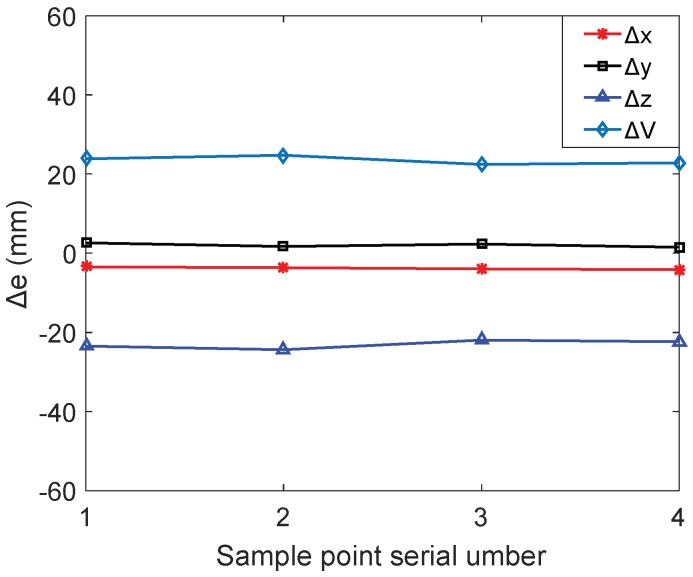
The position errors of the each optimal position before calibration.

In the motion control that is based on the initial measured kinematic parameters, the coordinate values of each optimal position are measured and the measured data are transformed by the mentioned method. The structural volume errors and the position errors of each optimal position are obtained as shown in Figure 11. It can be seen from Figure 11 that the error along the *z* axis is the main factor leading to the excessive position errors of the CDPR for 3D printing. Before calibration, the specific range of position errors and structural volume error of each optimal position are as shown in Table 6.

The position errors of the end-effector reflects the accuracy of the CDPR for 3D printing. According to the analysis of the data in Figure 11 and Table 6, it can be seen that the position errors along *z* axis are more than 20 mm. The accuracy requirement for 3D printing can not be satisfied due to the position errors. In order to solve this problem, the error identification should be implemented based on the above measurement data. Through compensating the initial measured kinematic parameter with identification results, the printing accuracy will be improved.

**Figure 12:**
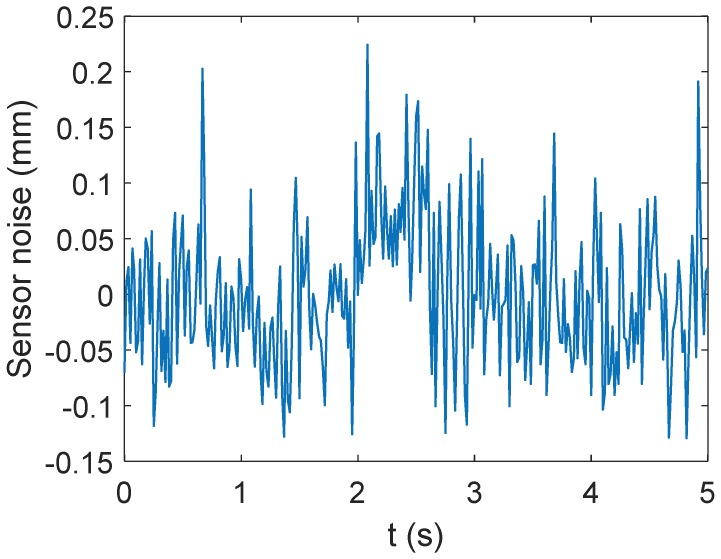
Measurement noise of the motion capture system.

**Figure 13:**
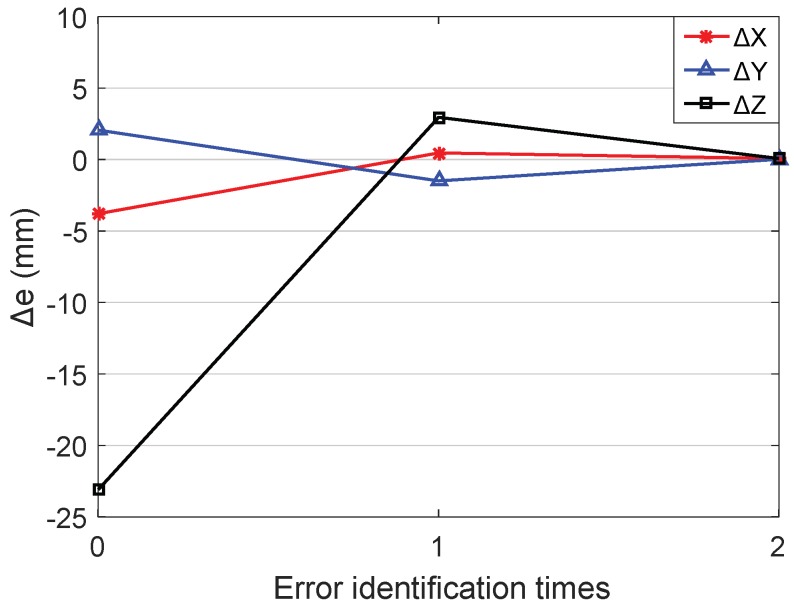
The relations between position errors and identification times.

In order to obtain valid error identification results, it is necessary to set a reasonable convergence threshold based on the measurement noise of the motion capture system. Through measuring the position coordinates of one fixed point repeatedly, the measurement noise of the motion capture system can be obtained. As shown in Figure 12, the fluctuation of the measurement noise is −0.12939 mm ≤$e_{k}$ ≤ 0.22407 mm. To reduce the influence of measurement noise on the error identification, a threshold as ξ = 0.80 mm is set. When the absolute value of the end-effector position errors meet the constraint |δ|≤ξ, the error identification cycle is terminated and the calibration is completed. After the parameter iteration, the position errors of the end-effector changing with the identification times are shown in Figure 13, after calibration, the errors of the end positions are as shown in Table 7. The identification errors of kinematic parameters are shown in Table 8.

From the above experimental results, one can obtain that the construction volume error of the end-effector position is reduced from 23.4662 mm to 0.4740 mm through the calibration. Among the position errors in each direction, the kinematic calibration is obviously effective to reduce the position errors along the *z* axis. The average errors in the z direction are reduced from −23.0636 mm to 0.3594 mm. Different from the position errors in the x and y directions, the position errors along the *z* axis will accumulate on the printed components as the printing layers increases for the fused deposition modeling. The proposed calibration method can bring obvious improvement of the position accuracy in the *z* direction and therefore, improve the printing accuracy of the CDPR for 3D printing effectively.

### 6.3. Calibration Result Verification

The calibrated kinematic parameters are applied to the control system, and the end-effector is controlled to move in four planes: Z = 175 mm, Z = 195 mm, Z = 215 mm, and Z=235 mm. On each plane, the ideal motion curve of the end-effector is the straight line from (20, 20) to (−20, −20). The static position errors of points on the line are measured to verify the effect of kinematic calibration. The measurement positions along the line are as follows: $M_{0}$(−20, −20), $M_{1}$(−16, −16), $M_{2}$(−12, −12), $M_{3}$(−8, −8), $M_{4}$(−4, −4), $M_{5}$(0, 0), $M_{6}$(4, 4), $M_{7}$(8, 8), $M_{8}$(12, 12), $M_{9}$(16, 16), and $M_{10}$(20, 20). The static position errors measurement is carried out to eliminate the influence of delay of the controller, actuator, spring, etc. In the static case, the error of the end-effector mainly comes from the error of kinematic parameters.

**Figure 14:**
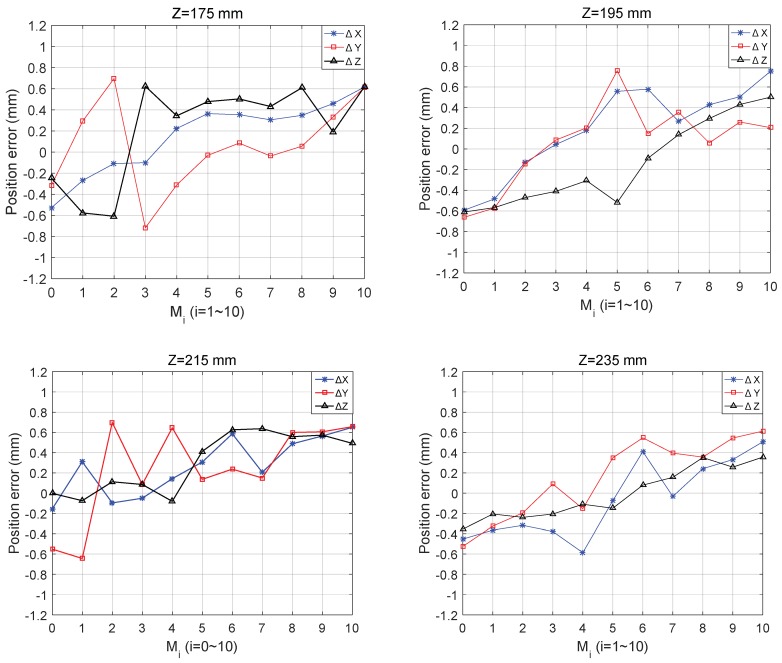
The error distributions of the end-effector after calibration.

One can obtain from Figure 14 that the measured positions covers four planes with different heights and the position errors of the calibrated CDPR for 3D printing in each direction of the end-effector remains within −0.80 mm ≤ δ ≤ 0.80 mm. The measurement results indicate that the proposed calibration method is effective and verified for measurement positions outside optimal positions set.

## 7. Conclusions

This paper presents a CDPR for 3D printing, which can improve the workspace of 3D printing device at a lower manufacturing cost, due to the extension range and flexibility of cables. 

In order to solve the kinematic calibration problem of CDPR for 3D printing, a kinematic calibration method based on cable length residuals is proposed. The functional formula is established between the measured information of the motion capture system and the actual kinematic parameters. According to the structure characteristics of the CDPR for 3D printing, a coordinate system conversion method for data measurement of the motion capture system is proposed, which provides the guarantee for obtaining accurate measurement data. The accuracy and effectiveness of this calibration method are verified though simulation. Furthermore, the kinematic calibration experiment is carried out on the basis of synchronous measurement of end-effector position errors and cable length variables with motion capture system. 

In order to further improve the efficiency of calibration and measurement, an optimal selection scheme for measurement positions is proposed. The simulation results of error modeling and analysis indicate that the accuracy of CDPR for 3D printing increases with printing height, and the maximum error on each horizontal plane is distributed close to the boundary of the workspace. The calibration experiments are carried out based on the optimized positions set, and the construction volume error of the end-effector is reduced from 23.4805 mm to 0.6157 mm. In addition, the proposed calibration method is effective and verified for measurement positions outside optimal positions set through experiments.

## Figures and Tables

**Table 1:** Coordinates of the optimal measurement positions.

	*x*/mm	*y*/mm	*z*/mm
$q_{1}$	−30.2310	24.5370	110
$q_{2}$	−42.5000	24.5370	110
$q_{3}$	−31.8320	43.015	110
$q_{4}$	−36.3660	13.9120	110

**Table 2:** The simulation results of the kinematic calibration.

Geometric Errors	Preliminary Errors (mm)	Error Identification Values (*i* = 1, 2, 3, 4)/(mm)			
		1	2	3	4
Δ*x*_1*a*_	2.0000	2.3485	−0.3485	${1.9536\;\times \;10}^{-8}$	$-{0.9257\;\times \;10}^{-8}$
Δ*y*_1*a*_	−1.1110	−1.3170	0.2060	$-{2.9026\;\times \;10}^{-9}$	${3.0060\;\times \;10}^{-9}$
Δ*z*_1*a*_	−1.0000	−1.0225	0.0225	$-{1.4291\;\times \;10}^{-9}$	${9.5420\;\times \;10}^{-10}$
Δ*x*_2*a*_	3.0000	3.2568	−0.2568	$-{3.2210\;\times \;10}^{-10}$	${0.0950\;\times \;10}^{-9}$
Δ*y*_2*a*_	−2.1110	−1.9264	−0.1845	${1.0411\;\times \;10}^{-10}$	$-{2.1103\;\times \;10}^{-10}$
Δ*z*_2*a*_	1.0000	1.0648	−0.0648	${2.5231\;\times \;10}^{-7}$	${1.4530\;\times \;10}^{-9}$
Δ*x*_3*a*_	0.0000	−0.1376	0.1376	${6.4737\;\times \;10}^{-8}$	${1.9241\;\times \;10}^{-9}$
Δ*y*_3*a*_	−0.7780	−1.9027	1.1245	${2.0310\;\times \;10}^{-4}$	${8.4712\;\times \;10}^{-10}$
Δ*z*_3*a*_	−0.5000	−0.2944	0.2055	$-{2.0476\;\times \;10}^{-5}$	$-{4.8903\;\times \;10}^{-7}$
Δ*l*_1_	2.0000	2.4042	− 0.4042	$-{4.2842\;\times \;10}^{-7}$	${2.2291\;\times \;10}^{-7}$
Δ*l*_2_	1.0000	0.1776	0.3223	$-{5.9930\;\times \;10}^{-10}$	${6.5831\;\times \;10}^{-10}$
Δ*l*_3_	3.0000	1.8487	1.1509	${2.0394\;\times \;10}^{-4}$	${1.5926\;\times \;10}^{-7}$

**Table 3:** The initial measurement of kinematic parameters.

Kinematic Parameters	Parameters without Calibration (mm)
A group cable length	382.0
B group cable length	387.0
C group cable length	388.0
The cable outlet A1	(306.0, −149.5, 77.0)
The cable outlet A2	(282.5, −188.5, 78.0)
The cable outlet B1	(−305.2, −149.1, 81.0)
The cable outlet B2	(−281.6, −189.7, 81.0)
The cable outlet C1	(−23.5, 339.0, 75.0)
The cable outlet C2	(23.5, 338.6, 77.0)

**Table 4:** The initial measurement of the virtual cable outlets.

Virtual Cable Outlets	Parameters without Calibration (mm)
A	(253.90, −146.50, 77.5)
B	(−253.30, −146.25, 81)
C	(0, 292.50, 76)

**Table 5:** The coordinate values of the markers on the base.

Markers on the Base	Coordinate Value (mm)
F1	(547.7330, 160.4733, 24.0144)
F2	(362.9547, −147.6336, 22.0150)
F3	(716.6375, −158.7557, 23.4959)

**Table 6:** The Error distribution area of the end-effector before calibration.

	Δx	Δy	Δz	ΔE
Maximal value	−3.4567	2.5909	−21.9312	22.3526
Minimum value	−4.1500	1.5527	−24.4203	24.8190
Average value	−3.8023	2.0687	−23.0636	23.4662

**Table 7:** Error distribution area of the end-effector after calibration.

	Δx	Δy	Δz	ΔE
Maximal value	0.4251	0.2145	0.4146	0.6314
Minimum value	−0.1354	−0.4783	0.3427	0.6038
Average value	0.2674	−0.1547	0.3594	0.4740

**Table 8:** The experimental results of kinematic calibration.

Kinematic Parameters	Before Calibration (mm)	Error Identification Value (*l* = 1, 2) (mm)		After Calibration (mm)
		First Time	Second Time	
$x_{1a}$	253.9000	2.07820	1.16230	257.1405
$y_{1a}$	−146.5000	−1.25331	−0.68765	−148.4410
$z_{1a}$	77.5000	1.85782	−0.46523	78.8926
$x_{2a}$	−253.3000	−3.31530	−0.40253	−257.0180
$y_{2a}$	−146.2500	−1.87986	−0.32561	−148.4550
$z_{2a}$	81.0000	−1.72365	−0.25632	79.0201
$x_{3a}$	0.0000	−0.12250	0.12585	0.0033
$y_{3a}$	292.5000	5.71754	0.53260	298.7501
$z_{3a}$	76.0000	4.66235	−2.18561	78.4767
$l_{1}$	382.0000	1.60682	1.26320	384.8700
$l_{2}$	387.0000	−2.65896	0.54825	384.8893
$l_{3}$	388.0000	−4.85620	2.15421	385.2980
